# Contribution of hip joint proprioception to static and dynamic balance in cerebral palsy: a case control study

**DOI:** 10.1186/1743-0003-10-57

**Published:** 2013-06-15

**Authors:** Diane L Damiano, Jason R Wingert, Christopher J Stanley, Lindsey Curatalo

**Affiliations:** 1 Functional & Applied Biomechanics Section Rehabilitation Medicine Department, National Institutes of Health, Room 1-1469, Bethesda, MD 20892, USA; 2 Department of Health & Wellness, University of North Carolina Asheville, WHC, CPO #2730 One University Heights, Asheville, NC 28804, USA

**Keywords:** Static balance, Sensory function, Sroprioception, Hemiplegia, Diplegia

## Abstract

**Background:**

Balance problems are common in cerebral palsy (CP) but etiology is often uncertain. The classic Romberg test compares ability to maintain standing with eyes open versus closed. Marked instability without vision is a positive test and generally indicates proprioceptive loss. From previous work showing diminished hip joint proprioception in CP, we hypothesized that static and dynamic balance without vision (positive Romberg) would be compromised in CP.

**Methods:**

Force plate sway and gait velocity data were collected using 3D motion capture on 52 participants, 19 with diplegic CP, 13 with hemiplegic CP, and 20 without disability. Center of mass (COM) and center or pressure (COP) velocity, excursion, and differences between COM and COP in AP and ML directions were computed from static standing trials with eyes open and closed. Mean gait velocity with and without dribble glasses was compared. Hip joint proprioception was quantified as the root mean square of magnitude of limb positioning errors during a hip rotation task with and without view of the limb. Mixed model repeated measures analysis of variance (ANOVA) was performed with condition as within-subject (EO, EC) and group as between-subject factors (hemiplegia, diplegia, controls). Sway characteristics and gait speed were correlated with proprioception values.

**Results:**

Groups with CP had greater sway in standing with eyes open indicating that they had poorer balance than controls, with the deficit relatively greater in the ML compared to AP direction. Contrary to our hypothesis, the decrement with eyes closed did not differ from controls (negative Romberg); however, proprioception error was related to sway parameters particularly for the non-dominant leg. Gait speed was related to proprioception values such that those with worse proprioception tended to walk more slowly.

**Conclusions:**

Postural instability is present even in those with mild CP and is yet another manifestation of their motor control disorder, the specific etiology of which may vary across individuals in this heterogeneous diagnostic category.

## Background

Cerebral palsy (CP) is the most common physical disability originating in childhood. Persons with cerebral palsy (CP) tend to have poorer static stability than those without CP [1,2]. However, mechanisms underlying balance disorders in CP are still not well understood. The Romberg test is a common neurological assessment for determining whether balance deficits are central (e.g. cerebellar) or peripheral (e.g. loss of position sense in the lower extremities) in nature by evaluating performance with eyes open and closed [3]. Markedly increased sway evident by clinical observation or falling with eyes closed compared to eyes open in quiet stance and feet close together is a positive test and generally indicates dorsal column or proprioceptive loss [4]. The primary goals of this study were to quantify responses to this test in patients with diplegic and hemiplegic CP compared to age-related controls, to characterize static and dynamic balance function in each CP sub-group, and to relate balance impairments to proprioceptive abilities in CP. Since our previously published data showed diminished large joint proprioception in CP, specifically for hip rotation [5], we hypothesized that both groups with CP would show a greater decrement in performance than controls with eyes closed. We further hypothesized that those who showed a greater decrement in balance performance with eyes closed or vision obscured in standing or walking, respectively, would tend to have poorer scores on proprioception tests, as well. The current study was part of a larger investigation of sensory function in children and young adults with unilateral and bilateral cerebral palsy compared to participants within the same age range with no neurological deficits [5-7].

Several studies have evaluated standing balance in spastic CP with most studies evaluating performance in groups with either diplegia only or groups that contained those with diplegia and hemiplegia. No study has evaluated static balance in those with hemiplegic CP alone, although dynamic stability during gait was investigated in CP subtypes [8]. Ferdjallah and coauthors [9] evaluated postural control synergies in 11 children with spastic diplegia, ages 6–18 years, and 8 children without disability, ages 5–13 years. They quantified sway separately in anterior –posterior (AP) and medial-lateral (ML) directions based on previous evidence supporting the fact that an ankle strategy is typically utilized for AP balance and a hip strategy is typically utilized for ML balance [10]. They found that the largest contributor to ML balance in all children was transverse body rotation and this was proportionately even greater for those with CP perhaps as a consequence of and compensation for their poorer ankle control. All children showed a similar reliance on visual input to aid stability regardless of group.

The largest static balance study to date by Rose and colleagues^2^ included 23 children with spastic diplegia ages 5–18 years compared to 32 children without disability in the same age range. Mean results indicated that the group with CP had increased displacement (sway) with eyes open and lower sway frequency despite the fact that the majority of children in the CP group had values within the normal range for most parameters. They also found that performance decrements with the eyes closed were similar across groups, suggesting that the group with CP did not have an increased need for visual feedback to maintain balance or, conversely, did not have a clinically significant loss of position sense. Cherng and coauthors [11] found that children with mild CP did not differ significantly from controls in their static balance abilities, but when under altered sensory conditions, clear decrements were noted compared to controls. Donker and colleagues [1] compared static balance in a group of 10 children with mixed types of CP to that of a group of 9 typically developing children, all within the range of 5–11 years of age. Children were asked to stand quietly wearing their typical orthotic devices and shoes with their eyes open, eyes closed and with real time feedback of their center of pressure. Similar to Rose and colleagues [2], they found that children with CP had greater sway magnitude than those without CP, which again was not shown to increase more in CP with eyes closed.

Postural abilities during static standing has been shown to be similar to those during more dynamic testing conditions in CP [12], although the more dynamic conditions may have greater sensitivity for identifying more subtle deficits or perhaps for elucidating potential mechanisms underlying decreased stability. Liao and colleagues[12] evaluated the relationship of clinical, static and dynamic posturography balance measures to gross motor functioning as assessed by the Motor Age Test in 15 children with various types of CP, ranging in age from 5–12 years. Motor Age was related to performance on instrumented eyes open, eyes closed and swaying vision static tests as well as two clinical tests, with those who were less functional having greater sway.

In summary, studies in CP generally, but not equivocally, report poorer balance in children with CP. Static balance has been shown to be related to dynamic stability in gait and to general motor ability in this population. The relationship of sensory function to motor performance in CP is not well studied or understood. This is the first study to relate quantitative lower extremity proprioception tests to static balance and gait performance in this population.

### Study design and methods

This was a prospective case control study that was approved by the institutional review board at Washington University School of Medicine. Written informed consent was obtained from all participants or their legal guardian with assent additionally obtained on those not old enough to provide consent.

Fifty-two children and young adults participated in this study, including 19 with diplegic CP (mean age (age range)): 14.6 years (7.3 – 34.3 years)), 13 with hemiplegic CP (13.1 years (7.3 – 23.4)), and 20 without disability (15.2 years (7.5 – 24.3)). All participants with CP were Gross Motor Functional Classification System (GMFCS) Levels I or II indicating that they had mild mobility impairments and were able to stand and walk without external assistance. Participants also needed to be able to follow complex instructions required for some of the sensory discrimination tasks. Exclusion criteria included diagnosis of athetoid or quadriplegic CP, previous selective dorsal rhizotomy, orthopedic surgery within the year prior to testing, botulinum toxin in the 6 months prior to testing, or marked visual or inner ear impairment. A modified Edinburgh Handedness Inventory [6,13] determined limb dominance; dominant leg was assumed to be ipsilateral [6]. In all participants with CP, the dominant side was the less impaired side, as assessed by responses to the modified Edinburgh Handedness Inventory. Table 1 displays the demographic characteristics of the three study groups.

**Table 1:** Demographics for all participants, diplegia (D), hemiplegia (H), and control (C)

	**Age**	**GMFCS**^**a**^	**MACS**^**b**^	**EHI**^**c**^	**Gestation age**	**Birth complications/**
						**probable CP etiology**
D3	23y 9 m	I	I	100	28 wks	grade III IVH, PVL
D4	28y 11 m	I	I	22.7	29 wks	PVL
D5	8y 10 m	I	I	85	35 wks	gestational diabetes
D6	14y 11 m	I	I	13.6	full term	unknown
D7	16y 5 m	I-II	I	20	23 wks	grade III IVH, PVL
D6	13y 7 m	I	I	95	26 wks	grade III IVH, PVL
D9	13y 6 m	I	I	90	30 wks	prematurity
D11	14y 0 m	I	I	95	28 wks	perinatal respiratory distress
D12	17y 0 m	I	I	90.9	31 wks	prematurity, twin, abnormal MRI
D13	13y 0 m	I	I	90	24 wks	grade III-IV IVH, PVL
D14	12y 7 m	I	I	100	26 wks	PVL
D15	8y 9 m	II	I	94.4	25 wks	PVL, twin
D16	10y 0 m	I	I	80	28 wks	PVL, twin
D17	9y 9 m	I-II	I	100	26 wks	grade IV IVH, PVL
D18	11y 8 m	I	I	95	31 wks	PVL, twin
D19	8y 9 m	I	I	10	33 wks	prematurity, triplet, placental abruption
D20	9y 9 m	I	I	5.6	26 wks	prematurity
D21	7y 4 m	I	I	0	31 wks	prematurity, twin
D23	34y 3 m	I	I	22.7	26 wks	prematurity
H1	8y 11 m	I	II	100	33 wks	perinatal right MCA stroke
H2	7y 4 m	I	II	100	N/A	perinatal right MCA stroke
H3	12y 10 m	I	I	100	36 wks	perinatal right MCA stroke
H4	10y 7 m	I	II	15	full term	perinatal left MCA stroke
H5	23y 5 m	I	II	100	full term	perinatal right MCA stroke
H6	13y 0 m	I	I	100	full term	postnatal right basal ganglia stroke
H7	13y 6 m	I	I	0	full term	perinatal left MCA stroke
H8	17y 4 m	I	I	13.6	26 wks	prematurity
H9	11y 1 m	I	II	0	36 wks	postnatal left MCA stroke
H10	18y 9 m	I	II	95.5	full term	left arachnoid cyst, right PVL, twin
H11	11y 9 m	I	I	100	25 wks	perinatal infection
H13	8y 7 m	I	I	0	full term	perinatal left MCA stroke, twin
H14	12y 10 m	I	I	0	full term	postnatal left MCA stroke
C1	11y 1 m			90.9	full term	
C2	10y 9 m			0.09	full term	
C3	18y 1 m			100	full term	
C4	8y 11 m			95.5	full term	
C5	24y 4 m			95.5	full term	
C6	15y 7 m			86.4	full term	
C7	24y 1 m			86.4	full term	
C8	9y 4 m			95.5	full term	
C9	16y 0 m			100	full term	
C10	7y 6 m			100	full term	
C11	14y 11 m			27.3	full term	
C12	11y 4 m			95.5	full term	
C13	23y 6 m			90.9	full term	
C14	9y 9 m			100	full term	
C15	15y 7 m			61.9	full term	
C16	13y 1 m			100	full term	
C17	15y 7 m			95.5	full term	
C18	18y 11 m			95.5	full term	
C19	18y 0 m			90.9	full term	
C20	17y 4 m			77.3	full term	

^a^Gross Motor Function Classification Scale (GMFCS).

^b^Manual Ability Classification System (MACS).

^c^modified Edinburgh Handedness Inventory (EHI).

The hip joint proprioception protocol has been described previously [6]. For brevity, hip proprioception was assessed using a custom built device that allowed rotation around the axis of a semi-goniometer, thereby measuring hip orientation angles in the transverse plane, more specifically hip internal and external rotation [6]. During testing, the participant was in supine with the upper trunk elevated ~45° on a wedge and the foot and lower calf were placed in a foam lined holder that accommodated variable limb sizes and minimized tactile cues. Participants were first asked to actively point their second toe at each of 10 target angles along the semi-goniometer axis, presented in a random order, with both their foot and the target fully visible. Then for 10 trials, the view of the limb was obscured by an opaque curtain and only targets on the goniometer were visible, requiring somatosensory input for limb guidance to complete the task. Participants performed the vision condition first to facilitate instruction and assess their motor abilities with respect to performing the task. For each limb and both conditions, the magnitude (degrees) of error between performance and target location were recorded for each trial to the nearest degree. Root mean square (RMS) of proprioception error was calculated for both the dominant and non-dominant legs. For each trial, error from the vision condition was subtracted from error for the no-vision condition to reflect proprioceptive contribution to the task. Proprioception errors from both legs were averaged yielding single values reflecting each participant’s total proprioception error for analysis.

To measure postural sway subjects stood barefoot with their feet side-by-side as close together as possible (@ 3 cm) without contacting the edges of two adjacent force plates (Kistler, Winterthur, Switzerland) and with arms relaxed at their sides. Participants performed three 20-second standing trials with eyes open (EO) and eyes closed (EC) and were instructed to remain as still as possible. All participants were able to stand with feet flat on the force platform, except for one participant with hemiplegia who was not able to achieve this position on the non-dominant side. Full body kinematic and kinetic data were captured with an 8-camera Vicon 612 System (Lake Forest, CA). This system tracked the movement of thirty-four reflective markers attached to anatomic landmarks (4 on head, C7, clavicle, sternum, T10, sacrum, right back, bilateral acromion, lateral elbow, ulnar styloid, radial styloid, hand, ASIS, thigh, lateral knee, shank, lateral ankle, heel, toe) at a sampling frequency of 120 Hz. Force plate data were collected at 1080 Hz.

Center of mass (COM) and center of pressure (COP) data were obtained from two separate software packages. The marker trajectory data were processed using a Woltring filter (predicted MSE value =15) in Vicon Nexus. Marker data and anthropometric measurements entered in Vicon were then used to create a subject-specific anatomical model from which the COM was calculated in Vicon Nexus. COP was calculated from force plate data that were filtered with a bidirectional low pass Butterworth filter (cutoff frequency 6 Hz) in Visual3D (C-Motion, Inc., Gaithersburg, MD). The average center of both feet in the medial-lateral (ML) and anterior-posterior (AP) directions for each trial was calculated in Visual3D.

Filtered COM and COP trajectories and feet center locations were exported to a custom-written Matlab program for additional processing and analysis. Applying the concept that the mean COP and COM in the ML and AP direction should be coincident, an offset was computed and applied to the COM time series data. The following variables were analyzed for both eyes open (EO) and eyes closed (EC): COM and COP velocity and excursion, and the differences between COM and COP in AP and ML directions. The mean value of the time series for each trial was calculated. The average of the EO trials and the EC trials was then calculated to have one representative value for each condition. The average COP location in the ML direction was determined to quantify sway asymmetry.

Subjects were also asked to walk barefoot at their self-selected speed on a 25 foot carpeted path with and without ‘dribble’ glasses that blocked the subject’s view of their feet, as a dynamic analogue to the static Romberg test. Five trials were collected per condition using the same marker set, motion capture system and sampling frequency as described above. Walking velocity was calculated for each trial using the trajectory data and then averaged for the conditions with and without dribble glasses.

Statistical analysis was performed in SPSS version 15 (SPSS Inc., Chicago, IL) with the level of significance at p < 0.05. A repeated measures analysis of variance (ANOVA) was conducted on the aforementioned dependent variables with condition (EO, EC) as the within-subjects factor and group (hemiplegia, diplegia, controls) as the between-subjects factor. A second analysis used dominance (dominant, non-dominant) as the within-subjects factor and group as the between-subjects factor to test for difference between dominant and nondominant sides within each condition (EO, EC). Paired or unpaired t-tests where performed for post hoc testing where indicated. An analysis of covariance (ANCOVA) was performed with the initial condition (EO) as the covariate to examine if groups performed significantly different during the eyes closed condition when controlling for the eyes open condition. A similar analysis was done comparing gait velocity with and without the dribble glasses.

Two-tailed Mann–Whitney U tests determined group differences in hip proprioception error, including within group (between sides) comparisons. For these tests, the significance level was Bonferroni-corrected for five comparisons per leg (each leg compared to side with same dominance in other two groups and to the contralateral leg within a subject), so that p ≤ 0.01. Pearson *r* correlation procedures were used to evaluate relationships among EO-EC differences in ML and AP directions for COM-COP, dominant, non-dominant, and total (averaged) hip proprioception errors, freely selected gait velocity without glasses as a measure of gait function and the difference in gait velocity with and without glasses.

## Results

**Figure 1:**
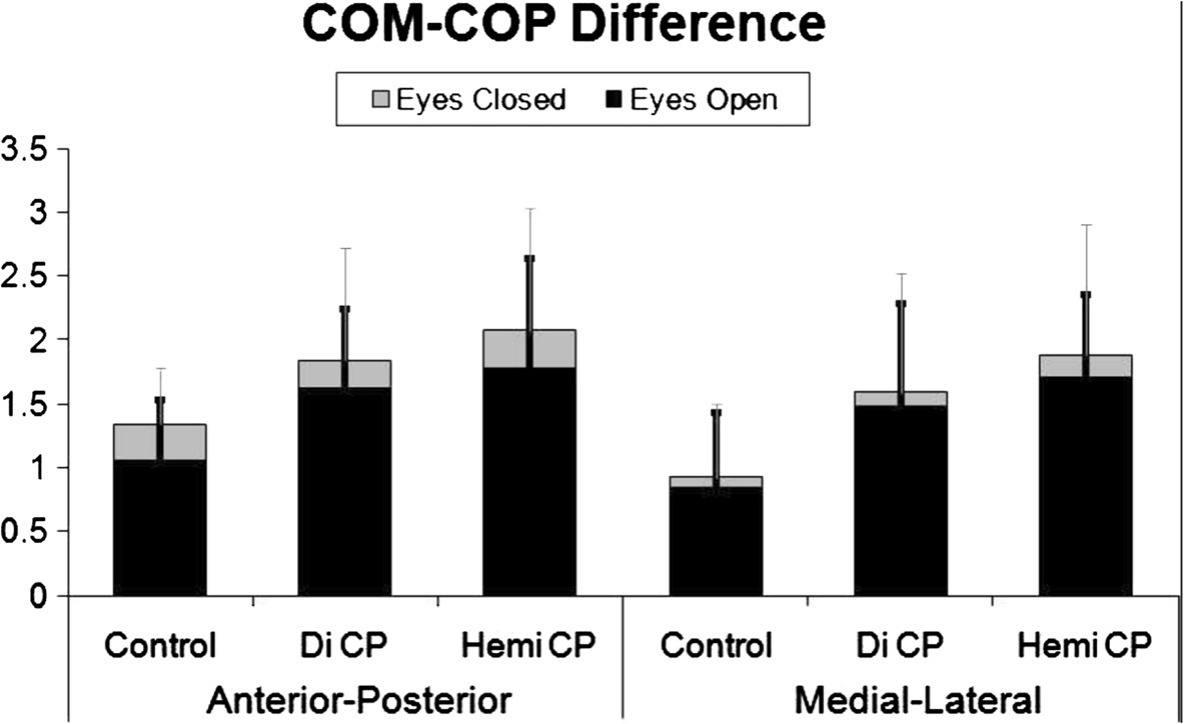
**Mean Stability Values by Group.** Mean Center of Mass – Center of Pressure (COM-COP) differences in mm with standard deviation of each indicated by error bars for each of the three groups in both directions with the eyes open and closed. DiCP = diplegic cerebral palsy and HemiCP = hemiplegic cerebral palsy.

Age was not significantly different across the three groups (p > 0.05; Table 1). Proprioception error was significantly higher in the two groups with CP when compared to controls on both the dominant and non-dominant hips and when averaged across sides (p < 0.01; Table 2). In EO and EC conditions, both groups with CP had larger COM and COP excursions and COM-COP differences (Figure 1; Table 3) in the AP & ML directions compared to controls. The velocity of sway for each condition and direction was significantly faster than controls only for hemiplegia but not diplegia, although the *p* values in the latter group ranged from 0.062-0.065 in the ML direction with consistently higher mean values as well suggesting a similar trend in that sub-group. Mean values for balance parameters by group are listed in Table 4. Gait velocity was significantly slower in the two groups with CP compared to controls (p < 0.05; Table 4).

**Table 2:** Comparison of Hip proprioception error across groups

**Hip proprioception error**	**Control**	**Diplegia**	**Hemiplegia**
**Dominant leg**			
RMS (degrees)	4.44	8.27*	11.52*
(95% CI)	(3.61 to 5.26)	(7.03 to 9.52)	(8.29 to 14.76)
**Non-dominant leg**			
RMS (degrees)	4.72	11.23*	12.90*
(95% CI)	(3.74 to 5.70)	(8.96 to 13.50)	(5.72 to 20.08)
**Average between legs**			
RMS (degrees)	4.58	9.75*	12.21*
(95% CI)	(3.84 to 5.31)	(8.34 to 11.16)	(7.37 to 17.06)

NOTE: Mann–Whitney U test significance levels were adjusted with the Bonferroni correction for 5 comparisons (*P* ≤ 0.01), and indicated where differences between group means are significant compared to the control group (*).

**Table 3:** Mean balance parameter values by group

**Gait condition**	**Control**	**Diplegia**	**Heimplegia**
Center of Mass (COM)			
EO range AP	13.82	23.41*	21.29*
(95% CI)	(11.1 to 16.52)	(18.89 to 27.94)	(16.54 to 26.04)
EO range ML	5.8	19.09*	17.22*
(95% CI)	(4.04 to 7.57)	(12.68 to 25.50)	(9.18 to 25.26)
EC range AP	15.59	26.13*	24.82*
(95% CI)	(11.33 to 19.85)	(20.61 to 31.65)	(17.95 to 31.70)
EC range ML	6.11	23.19*	23.73*
(95% CI)	(4.25 to 7.98)	(14.96 to 31.41)	(13.03 to 34.43)
Center of Pressure (COP)			
EO range AP	18.54	31.16*	30.09*
(95% CI)	(15.12 to 21.95)	(25.60 to 36.71)	(23.61 to 36.57)
EO range ML	10.63	25.89*	28.48*
(95% CI)	(7.63 to 13.63)	(16.76 to 35.01)	(14.30 to 42.66)
EC range AP	22.12	34.26*	35.6*
(95% CI)	(16.77 to 27.47)	(27.73 to 40.79)	(26.91 to 44.29)
EC range ML	11.58	31.13*	35.51*
(95% CI)	(7.82 to 15.34)	(19.51 to 42.75)	(20.62 to 50.40)

NOTE: Mann–Whitney U test significance levels are indicated where differences between group means are significant (p < 0.05) compared to the control group (*).

**Table 4:** Mean gait velocity by condition and by group

**Gait condition**	**Control**	**Diplegia**	**Hemiplegia**
**Gasses off**			
mean velocity (m/s)	1.29	1.10*	1.05*
(95% CI)	(1.21 to 1.37)	(1.03 to 1.17)	(0.90 to 1.21)
**Glasses on**			
mean velocity (m/s)	1.30	1.16*	1.04*
(95% CI)	(1.20 to 1.39)	(1.10 to 1.23)	(0.93 to 1.14)

NOTE: Mann–Whitney U test significance levels are indicated where differences between group means are significant (p < 0.05) compared to the control group (*).

The measure deemed here to be the quantitative representation of the Romberg test was the amount of increase in the COM-COP difference in both AP and ML directions from the EC condition, controlling for the EO condition, where a significantly greater increase over the control values were considered indicative of a positive test. These values are depicted in Figure 1. Groups did not perform differently during EC in either direction, indicating that the loss of vision produced a similar decrement in performance across groups. Therefore, the Romberg test was considered to be negative for both groups with CP. Similarly, the glasses - no glasses velocity when walking showed no differences across groups (Table 4).

**Figure 2:**
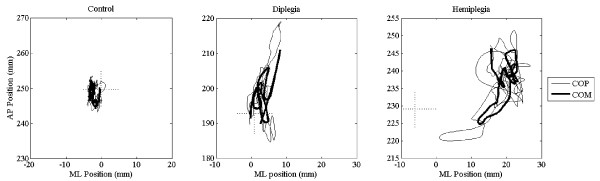
**Representative stabilogram for each group.** Center of Mass (COM) and Center of Pressure (COP) tracings show individual examples of increased anterior-posterior (AP) sway in diplegia and prominently increased medial-lateral (ML) sway more in hemiplegia compared to controls. Cross represents center between feet showing asymmetry in hemiplegia.

A difference between one of the CP groups and the other two groups was found when examining sway asymmetry. The group with hemiplegia was more asymmetric in stance with an average COP ML location of 17 mm off center towards the dominant side, compared to <1.5 mm for controls and diplegia (Figure 2).

Hip proprioception error was significantly correlated with balance parameters (Table 5) when tested across all participants. Relationships between proprioception and balance were strongest for proprioception in the non-dominant leg, where proprioception error was significantly correlated with all balance parameters (p < 0.05). For example, as proprioception error increased, COM sway with eyes closed also increased in the ML (EC range ML) direction (r = 0.42; p = 0.004; Table 5). However, these relationships were either non-existent or less robust when tested within groups. Lower extremity proprioception values were not related to EO-EC differences.

**Table 5:** Pearson correlation between proprioception error and balance parameters (n = 53)

	**Center of Mass (COM)**				**Center of Pressure (COP)**			
	**EO range AP**	**EO range ML**	**EC range AP**	**EC range ML**	**EO range AP**	**EO Range ML**	**EC range AP**	**EC range ML**
**Dominant leg**	0.28	0.20	0.24	0.27	0.31	0.17	0.27	0.22
Pearson r								
p-value	0.04	ns	ns	0.05	0.03	ns	ns	ns
**Non-dominant leg**								
Pearson r	0.55	0.41	0.42	0.40	0.55	0.38	0.45	0.36
p-value	< 0.0001	0.003	0.002	0.004	< 0.0001	0.005	0.0008	0.01
**Average between legs**								
Pearson r	0.44	0.32	0.35	0.36	0.46	0.29	0.39	0.31
p-value	0.001	0.02	0.01	0.008	0.0006	0.034	0.005	0.03

Mean proprioception error also had a significant negative correlation with gait velocity (Table 6) when tested across all participants (n = 53), such that those with higher error walked more slowly (*r* = −0.49; p = 0.0002). This negative correlation was also evident between proprioception and gait velocity for proprioception error in the dominant leg (*r* = −0.49; p = 0.0002) and less so in the non-dominant leg (*r* = −0.37; p = 0.006). However, correlations between proprioception error and gait velocity were not found within groups, except for one notable exception; in participants with hemiplegia there were significant negative correlations between proprioception error on the dominant side and gait velocity, both while wearing (r = −0.65; p = 0.01) and not wearing (r = −0.59; p = 0.03) dribble glasses. The glasses-no glasses difference was not related to the EO-EC difference, nor was it related to proprioception values.

**Table 6:** Pearson correlation between proprioception error and gait velocity (n = 53)

	**glasses off**	**glasses on**
**Dominant leg**		
Pearson r	−0.49	−0.43
p-value	0.0002	0.001
**Non-dominant leg**		
Pearson r	−0.37	−0.33
p-value	0.006	0.02
**Average between legs**		
Pearson r	−0.49	−0.43
p-value	0.0002	0.001

## Discussion

Three distinct sensory systems contribute to postural stability. Peterka and Loughlin [14] propose that while visual, somatosensory and vestibular inputs are each important for maintaining balance, their contribution or relative ‘weighting’ varies by task. Maintaining balance on an unstable surface relies primarily on vestibular input with somatosensation the least important. In contrast, static balance on a stable surface as measured in our study typically relies primarily on somatosensory input. Visual input can compensate for somatosensory deficits during this task, but when vision is removed, these may then become evident. This information further validates the use of the Romberg test as a simple and direct clinical measure of somatosensation.

Although the hallmark of CP is motor dysfunction, there is increasing evidence of alterations in sensory pathways in this population that may exceed those in the motor pathways as shown by Diffusion Tensor Imaging (DTI) studies [15]. Wingert and co-authors [7] recently reported diminished blood oxygen level dependent (BOLD) responses to sensory stimuli during fMRI in a subset of the participants with CP described in this study. Interestingly, each also performed a button push to identify the stimulus, and the BOLD response to that motor task did not differ between controls and those with diplegic CP. These fMRI results appear to corroborate the DTI findings in this population. Whether the damage in the sensory pathways is merely coincident with, or whether it may be a cause or consequence of the injury to the motor pathways or cortical areas remains unknown.

Children with CP do not lack somatosensation entirely in any body part as seen in many children with spina bifida or spinal cord injuries [16,17], but both tactile and proprioceptive tests reveal diminished performance even in children with CP who have mild to moderate functional motor involvement compared to controls [5,6,18]. Measurement of hip proprioception is particularly relevant in CP because they tend to rely disproportionately more on this joint in their balance strategies compared to those without CP [19,20]. Sensory function was shown to be moderately related to both unimanual and bimanual upper extremity performance measures in a group of 70 children with hemiplegic CP [18]. Similarly, we showed here that sensory and motor function are also related in the lower extremities, as seen by the correlation between proprioception error scores and both standing balance and gait. Increased hip proprioception error was related to increased postural sway and decreased gait velocity. This was especially true for participants with hemiplegia, whose dominant side hip proprioception error was an important determinant of gait velocity. Therefore, hip proprioception deficits accounted for some of the variance in balance and gait velocity performance both with and without vision, even in people with milder CP.

Skilled movements such as reaching can still be performed in the absence of somatosensation, using visual guidance as a compensation [20]. However, removing vision in this group that had proprioceptive loss did not significantly additionally disrupt standing or walking as hypothesized. This could be explained by the correlation results showing that lower extremity motor performance was directly related to somatosensation even with vision. It should be noted that correlations were stronger and more consistent without vision, suggesting that differences between vision and no vision conditions may become more apparent in those with greater sensory and/or motor impairments.

There is growing scientific interest in somatosensation because the integrity of this sensory system in CP may be a critical factor in the success of interventions that utilize sensory pathways to help drive motor recovery. It has been shown that children with hemiplegia who had greater sensory impairment did not respond as positively to motor training; however in that same study, sensory and motor function were directly related which means that the group that did worse was also more impaired motorically to start with [18]. Having intact sensation has also been mentioned as an inclusion criterion for pediatric constraint trials [21]. In addition to the growing research interest in using sensory input to stimulate motor improvement, evidence is also emerging to support that motor training can improve sensation [22].

Even though the Romberg test was negative here, children with CP in this study had poorer static stability with their eyes open compared to controls. Poor balance with eyes open may be indicative of cerebellar dysfunction in CP which is often suspected but has not been supported by sufficient evidence. Calberg and Hadders-Algra [23] identified inappropriate muscle coordination patterns during voluntary and involuntary activity as the major contributor to postural difficulties, but these patterns could be caused by injuries in many different central nervous system (CNS) regions so the underlying mechanism is still unclear. Other potential contributors to postural instability in CP include dystonia which is involuntary muscle activation when attempting to produce a voluntary movement or sustain a posture. Alternatively, some patients with CP may be cocontracting their muscles to compensate for weakness, which has been shown to explain a significant proportion of the variance in postural instability in this population [24]. Roncesvalles and co-investigators [25] found that the level of cocontraction was actually insufficient, rather than excessive as postulated in CP, when attempting to recover balance after a perturbation. Knowledge of the precise mechanisms underlying postural instability is critical for designing and prescribing the most effective treatment strategies.

Both groups with CP had greater differences from control values in the ML direction than in the AP direction. According to O’Connor and Kuo [26], balance control in the lateral direction requires more active motor control than the fore-aft direction. Since CP by definition is a disorder of motor control, it is not surprising that this directional difference exists. The only major dissimilarity between groups with CP was that the group with hemiplegia differed from both the control group and the group with diplegia by demonstrating significant asymmetry in the mean COP ML location with a consistent shift toward the dominant side. From a biomechanical standpoint, this asymmetry is likely to have marked secondary effects on muscle and bone development in both lower extremities and is consistent with differences that have been noted in this population such as decreased limb development and skeletal maturity on the hemiplegic side [27]. From a neurological standpoint, the continued greater reliance on one limb may also have a progressively negative effect on brain development as well since the level of ‘activity’ differs across sides. Intervening early in development to minimize these secondary effects clearly should be a high priority. Intense ‘forced use’ therapeutic strategies in the lower limb such as treadmill training may help diminish some of the asymmetry in the short term as has been shown in adults post-stroke [28,29]. These findings need to be replicated in CP and residual and longer term effectiveness should also be evaluated.

## Conclusions

In conclusion, individuals with mild unilateral and bilateral CP demonstrate balance deficits when attempting to stand still that does not worsen more than normally expected when their eyes are closed, even with documented evidence of diminished proprioception in many of the participants. However, those with better lower extremity proprioception values walked faster and had less postural sway, showing that a link does exist between sensory and motor performance in the lower as well as the upper extremities. The causes of balance deficits are likely multi-factorial in CP and need to be elucidated so that effective treatments can be designed. Plasticity in the sensory and motor systems and their reciprocal interactions are exciting new avenues for neurorehabilitation research that offer great promise for functional and brain recovery in those with CP and other CNS disorders and warrant further exploration.

## Abbreviations

ANOVA: Analysis of variance; BOLD: Blood oxygen level dependent; AP: Anterior-Posterior; COM: Center of Mass; COP: Center of pressure; CP: Cerebral palsy; EC: Eyes closed; EO: Eyes open; GMFCS: Gross motor functional classification system; ML: Medial-lateral.

## Competing interests

The authors declare that they have no competing interests.

## Authors’ contributions

JW, DD, and CS participated in data collection, JW, CS, AND LC processed the data, LC and JW developed and implemented new methods of data analyses for this manuscript, DD, JW, LC and CS contributed to statistical analyses and interpretation, DD, JW and LC drafted sections of the manuscript. DD and JW conceived of the study, and participated in its design and coordination. All authors read and approved the final manuscript.
